# Response of FGFR-2 Positive Adenoid Cystic Carcinoma to Futibatinib: A Case Report

**DOI:** 10.7759/cureus.63332

**Published:** 2024-06-27

**Authors:** Yoan E Rodriguez, Maham Shahid, Natalia Badillo, Augusto Villegas, Nilmarie Guzman

**Affiliations:** 1 Internal Medicine, Orange Park Medical Center, Orange Park, USA; 2 Internal Medicine, HCA Florida Orange Park Hospital, Orange Park, USA; 3 Hematology and Oncology, Florida Cancer Specialists and Research Institute, Fleming Island, USA

**Keywords:** salivary gland tumor, futibatinib, fibroblast growth factor inhibitor, fibroblast growth factor receptor (fgfr), adenoid cystic carcinoma (acc)

## Abstract

Adenoid cystic carcinoma (ACC) is an uncommon and aggressive head and neck cancer mainly affecting minor salivary glands. It affects more women than men in their 60s and 70s. The tumor is typically locally aggressive and has a high rate of distant metastatic disease. This report unveils a potential avenue for targeted therapy for the management of metastatic disease: a patient with ACC who harbored a specific fibroblast growth factor receptor 2 (FGFR-2) mutation and responded significantly to a novel FGFR-2 inhibitor. This finding could pave the way for personalized treatment options for ACC patients with similar genetic alterations. Nevertheless, the use of futibatinib requires further investigation to optimize treatment protocols, including exploring combination therapies, identifying predictive biomarkers for treatment response, and developing strategies to overcome potential resistance.

## Introduction

Adenoid cystic carcinoma (ACC) is a rare and aggressive epithelial malignancy that can arise in various head and neck locations, including the salivary glands, sinonasal cavity, oral cavity, and trachea. Although ACC represents only 1% of head and neck malignancies, it accounts for 10% of all salivary gland malignancies [1]. ACC exhibits a peak incidence between the sixth and seventh decades of life and is more common in women than men, as evidenced by a reported female-to-male ratio of 3:2 [1]. Surgery and adjuvant radiotherapy are the preferred treatment modalities for ACC [2]. However, these treatment modalities are associated with substantial morbidity and a high risk of recurrence and metastatic disease [3]. On the other hand, fibroblast growth factor receptors (FGFR) are receptor tyrosine kinases involved in multiple biological activities, including tissue development and repair, but receptor mutation can promote tumorigenesis [4]. Alterations in fibroblast growth factor receptor 2 (FGFR-2) have been linked to gastric cancer, intrahepatic cholangiocarcinoma, and endometrial uterine cancer [5]. 

Futibatinib is an irreversible, selective inhibitor of FGFR1-4 that forms a covalent cysteine residue in the P-loop structure of the FGFR kinase domain [6]. In patients with FGFR-2 mutant intrahepatic cholangiocarcinoma, futibatinib has demonstrated antitumor activity effectiveness and due to its irreversible binding mechanism, it is less vulnerable to on-target resistance mutations than other competing FGFR inhibitors such as pemigatinib and infigratinib [6]. Consequently, the Food and Drug Administration (FDA) has approved futibatinib for the treatment of intrahepatic cholangiocarcinoma containing FGFR2 mutations, which was previously treated and was unresectable, locally advanced, or metastatic [6]. Nonetheless, response to therapy for FGFR inhibitors has been variable in general, which highlights the need for further research to determine which FGFR-2 mutations could be therapeutic targets [7]. Additionally, FGFR-2 mutations have not been extensively identified as a potential therapeutic target for ACC. This case report presents a patient with ACC harboring an FGFR-2 mutation who experienced a significant response to futibatinib, a novel FGFR-2 inhibitor.

## Case presentation

A 69-year-old male with a past medical history of hypertension, hyperlipidemia, and active tobacco use presented to our oncology clinic for the management of recurrent left ear canal ceruminous ACC. Two years prior, he was evaluated by an otorhinolaryngologist for ear pain and swelling and was diagnosed with grade 3 ACC of the left external ear canal. He underwent surgical resection which included left-sided parotidectomy, condylectomy, auriculectomy, partial temporal bone excision, and neck lymph node dissection at that time. While all lymph nodes were negative, he had positive margins and received postoperative adjuvant radiation. 

**Figure 1:**
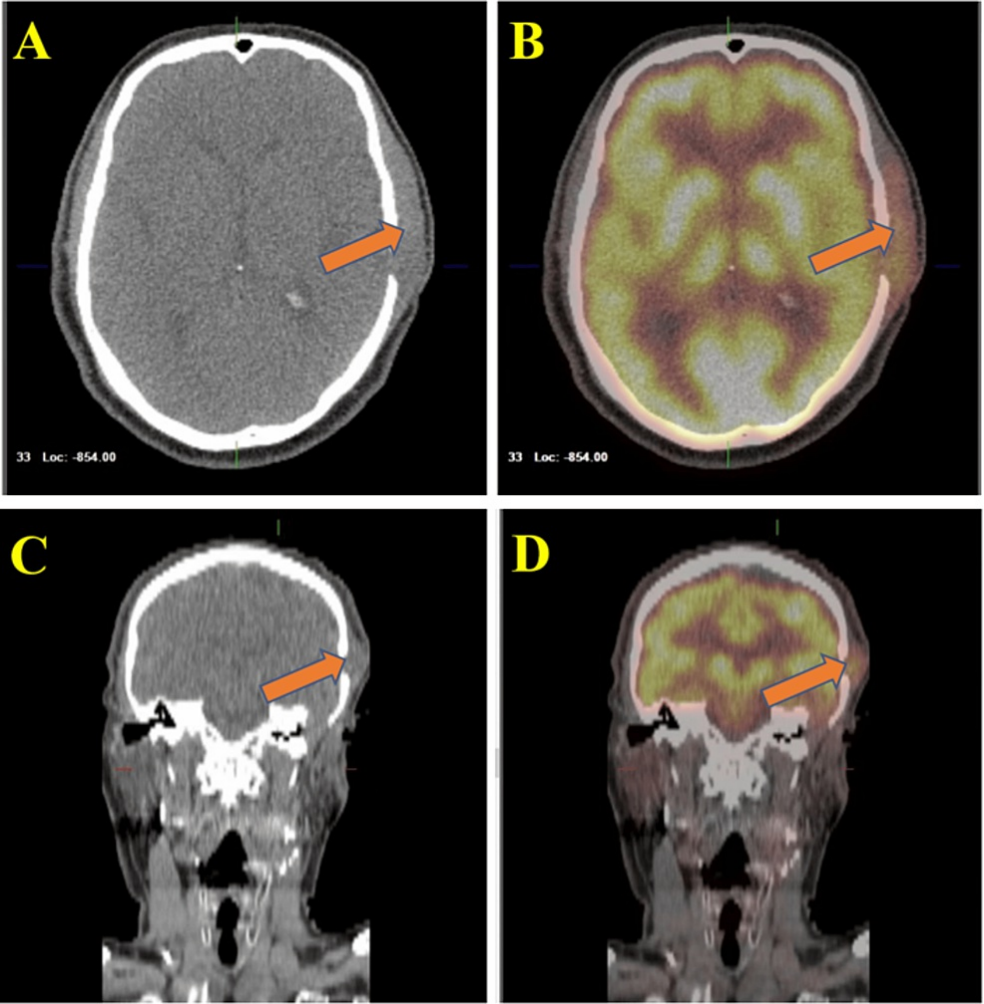
Pre-treatment positron emission tomography-computed tomography (PET-CT) scan of the head and neck Images A and B display coronal views, whereas images C and D show axial views of the PET-CT scan. This scan reveals a region of increased fluorodeoxyglucose (FDG) uptake along the left subcutaneous scalp, which is thickened and measures 6.3 cm in the anteroposterior dimension. The maximum standardized uptake value (SUV) noted is 8.7. There is no clear evidence of FDG avid adenopathy.

Subsequent recurrence of ear pain and swelling prompted further oncologic consultation. Positron emission tomography-computed tomography (PET-CT) scan from the skull base to mid-thigh demonstrated local recurrence of a left facial mass measuring 6.3 cm in the anteroposterior dimension without lymph node involvement as shown in Figure 1. Additional workup revealed recurrent ACC for which complementary tissue and circulating tumor DNA analysis were positive for FGFR-2 mutations.

**Figure 2:**
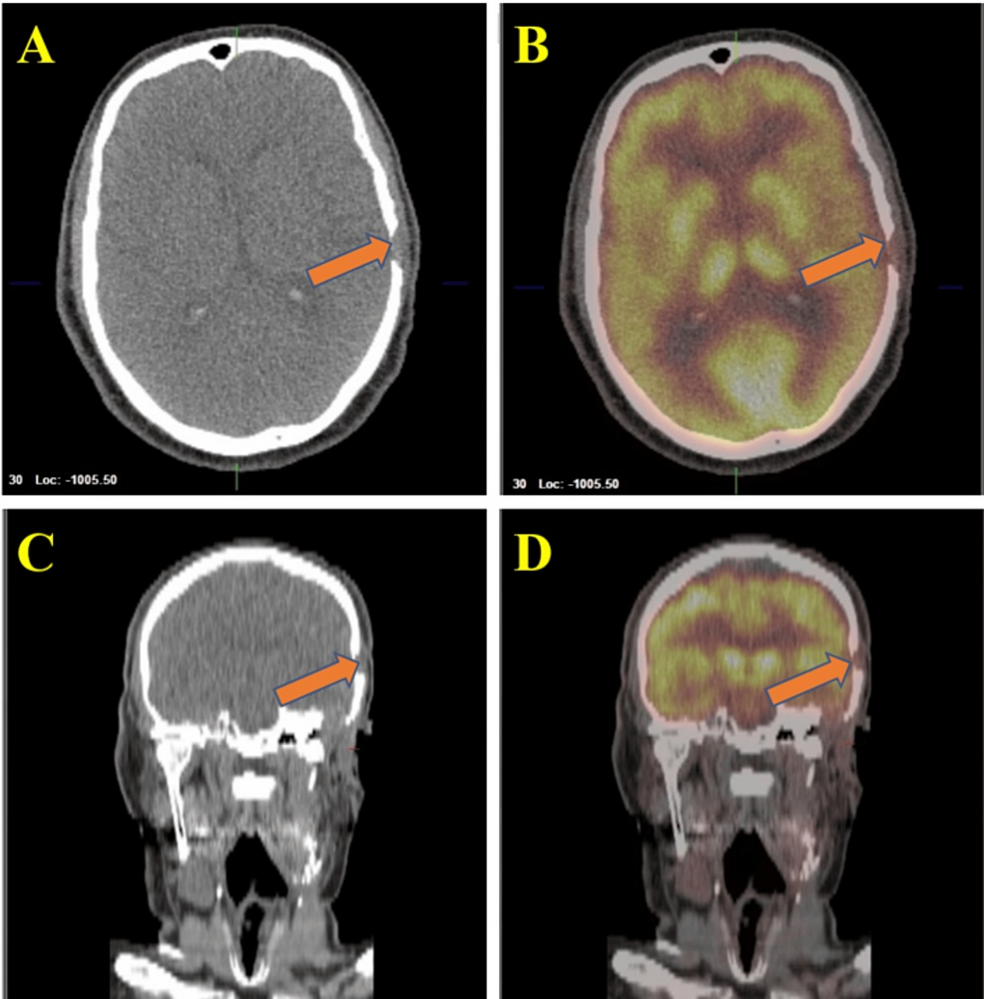
Post-treatment positron emission tomography-computed tomography (PET-CT) scan of the head and neck Images A and B display coronal views, whereas images C and D present axial views of a follow-up PET-CT scan. The scan indicates a significant reduction in soft tissue thickening in the left subcutaneous scalp, now showing an essentially physiological appearance on the CT images. The intensity reveals a maximum SUV of up to 3.7, in contrast to the previous maximum SUV of 8.7. Additionally, there is no evidence of fluorodeoxyglucose (FDG) avid adenopathy.

Treatment options were discussed including standard chemotherapy with cyclophosphamide, doxorubicin, and cisplatin versus off-label therapy with futibatinib since the tumor harbored an FGFR-2 mutation. After a discussion of risks, benefits, and potential side effects, a decision was made to initiate systemic therapy with futibatinib 20mg daily for a 21-day cycle. The patient responded extremely well with a near complete resolution of the mass on the left temple on physical examination. Repeat PET-CT scan performed after two cycles of futibatinib (around eight weeks after initial therapy) demonstrated notably decreased conspicuity of left subcutaneous scalp soft tissue as shown in Figure 2. The patient had only mild asymptomatic hyperphosphatemia resulting from therapy and no visual complications occurred. 

## Discussion

The most common initial treatment for ACC is surgery, either with or without radiation therapy [2,8]; nevertheless, most patients later develop metastases or tumor recurrences [8]. Currently, there is no standard chemotherapy for ACC due to its rarity and unclear molecular etiology, and many patients experience tumor recurrence and metastatic illness [9]. A common chemotherapy regimen for ACC involves cisplatin, doxorubicin, and cyclophosphamide (CAP), which can be combined with 5-fluorouracil [9]. While chemo plays a role in tackling aggressive ACC, the best drug combinations are still being figured out. This case highlights the potential of targeted therapy with futibatinib for patients with FGFR-2-mutated ACC. Therefore, a targeted therapy approach with futibatinib could potentially minimize unnecessary side effects associated with extensive surgery and radiation or additional traditional chemotherapy. However, further investigation is needed to optimize treatment regimens and confirm the efficacy and safety of futibatinib in a larger patient population. 

The rapid and significant tumor response observed in this patient suggests that futibatinib offers a promising therapeutic option for the management of ACC. We believe that by inhibiting FGFR-2 signaling, futibatinib directly attacked the driver mutation behind tumor growth. Therefore, identifying the specific FGFR mutations that predict sensitivity to futibatinib is crucial for optimal patient selection and ensuring treatment efficacy. Although the rapid tumor response to futibatinib in this particular case is encouraging, its long-term effectiveness and potential for resistance remain unclear. 

Futibatinib can cause hyperphosphatemia in 82% of patients and ocular toxicity such as retinal disorders (8%) and cataracts (4%) [6,10]. Therefore, frequent ophthalmology evaluation and monitoring of phosphate levels are recommended. The occurrence of nail disorders, hepatic toxicity, stomatitis, palmar-plantar erythrodysesthesia syndrome, and rash have been also linked to the use of futibatinib. A recent comprehensive safety analysis of this medication revealed a consistent and controllable safety profile among over 450 patients with different types of solid tumors [10]. The analysis indicated that the majority of adverse events were of mild or moderate severity, with rare instances of treatment-related discontinuations and no treatment-related fatalities. Moreover, most of these adverse events were effectively addressed through modifications in dosage and/or the implementation of supportive care interventions.

While we will continue to follow this patient to monitor the lasting benefits of futibatinib, further studies with larger patient cohorts are needed to confirm its efficacy and generalizability. On the other hand, exploring combinations of futibatinib with other therapies, such as immunotherapy or conventional treatments for non-responders, could potentially optimize treatment outcomes and improve patient care. 

## Conclusions

This case report not only presents a compelling example of the potential that futibatinib holds for treating FGFR-2-mutated ACC but also emphasizes the importance of genetic testing and patient autonomy in tailoring treatment strategies. The emergence of personalized medicine in oncology empowers clinicians to tailor treatment strategies based on a patient's unique tumor characteristics, maximizing treatment efficacy and minimizing side effects. Futibatinib therapy offers a potential paradigm shift in the management of ACC aggressive tumors, offering hope for improved clinical outcomes and personalized treatment options. Further research is required to decide long-term efficacy, optimize treatment regimens, and broaden patient selection. Moreover, continued efforts toward making this promising therapy accessible and affordable for all patients are essential to unlock its full potential in fighting FGFR2-positive ACC. 
